# Unexpected systolic anterior motion of the mitral valve-related hypoxemia during transurethral resection of the prostate under spinal anesthesia: a case report

**DOI:** 10.1186/s12871-022-01754-x

**Published:** 2022-07-06

**Authors:** Chien-Ju Chou, Yi-Chen Lai, Shu-Yu Ou, Chen-Hsiu Chen

**Affiliations:** 1 Department of Anesthesiology, Kaohsiung Armed Forces General Hospital, Kaohsiung, Taiwan; 2 Department of Anesthesiology, Kaohsiung Veterans General Hospital, No. 386, Dazhong First Road, Zuoying District, 81362 Kaohsiung City, Taiwan; 3 School of Medicine, National Yang Ming Chiao Tung University, Taipei, Taiwan; 4 National Sun Yat-sen University, Kaohsiung, Taiwan

**Keywords:** Pulmonary edema, hypoxia, Systolic anterior motion, Transesophageal echocardiography, Spinal anesthesia

## Abstract

**Background:**

Dynamic obstruction of the left ventricular outflow tract resulting from systolic anterior motion of the mitral valve can be an unexpected cause of acute and severe perioperative hypotension in noncardiac surgery. We report a patient undergoing spinal anesthesia for transurethral resection of the prostate who experienced sudden hypoxemia caused by systolic anterior motion-induced mitral regurgitation but with a clinically picture simulating fluid overload.

**Case presentation:**

An 83-year-old man with a history of hypertension was scheduled for transurethral resection of the prostate. One hour after spinal anesthesia, he developed acute restlessness and dyspnea, with pink frothy sputum and progressive hypoxemia. Slight hypertension was noted, and an electrocardiogram showed atrial fibrillation with a rapid ventricular response. Furosemide and nitroglycerin were thus administered for suspected fluid overload or transurethral resection of the prostate syndrome; however, he then became severely hypotensive. After tracheal intubation, intraoperative transesophageal echocardiography was promptly performed, which revealed an empty hypercontractile left ventricle, significant mitral regurgitation and mosaic flow signal in the left ventricular outflow tract. Following aggressive fluid therapy, his hemodynamic changes stabilized. Repeat echocardiography in intensive care unit confirmed the presence of systolic anterior motion of the anterior mitral leaflet obstructing the left ventricular outflow tract. We speculate that pulmonary edema was induced by systolic anterior motion-associated mitral regurgitation and rapid atrial fibrillation, and the initial management had worsened his hypovolemia and provoked left ventricular outflow tract obstruction and hemodynamic instability.

**Conclusions:**

Pulmonary edema caused by systolic anterior motion of the mitral valve can be difficult to clinically differentiate from that induced by fluid overload. Therefore, bedside echocardiography is paramount for timely diagnosis and prompt initiation of appropriate therapy in the perioperative care setting.

**Supplementary Information:**

The online version contains supplementary material available at 10.1186/s12871-022-01754-x.

## Background

Systolic anterior motion (SAM) of the mitral valve is defined as the anterior displacement of a mitral leaflet toward the left ventricular outflow tract (LVOT) during systole, causing LVOT obstruction and mitral regurgitation (MR) [1]. The typical manifestation is unexpected cardiovascular collapse, commonly seen in patients with hypertrophic cardiomyopathy (HCM) [2]. Notably, in the absence of underlying cardiac disease, SAM may also be triggered by increased catecholaminergic stimulation and reduction in ventricular preload, such as hypovolemia, anesthesia-mediated vasodilatation or neuraxial anesthesia [3, 4]. We present the case of a patient who developed acute hypoxemia caused by SAM-associated MR but without hypotension while undergoing transurethral resection of the prostate (TURP) under spinal anesthesia, and discuss the fundamental role of intraoperative transesophageal echocardiography (TEE) in diagnosis.

## Case presentation

An 83-year-old man (weight 60 kg, height 165 cm) was admitted to our hospital for laser TURP due to recurrent prostate adenoma. Past medical history included hypertension, peptic ulcer bleeding and C6–C7 disc herniation.

**Fig. 1:**
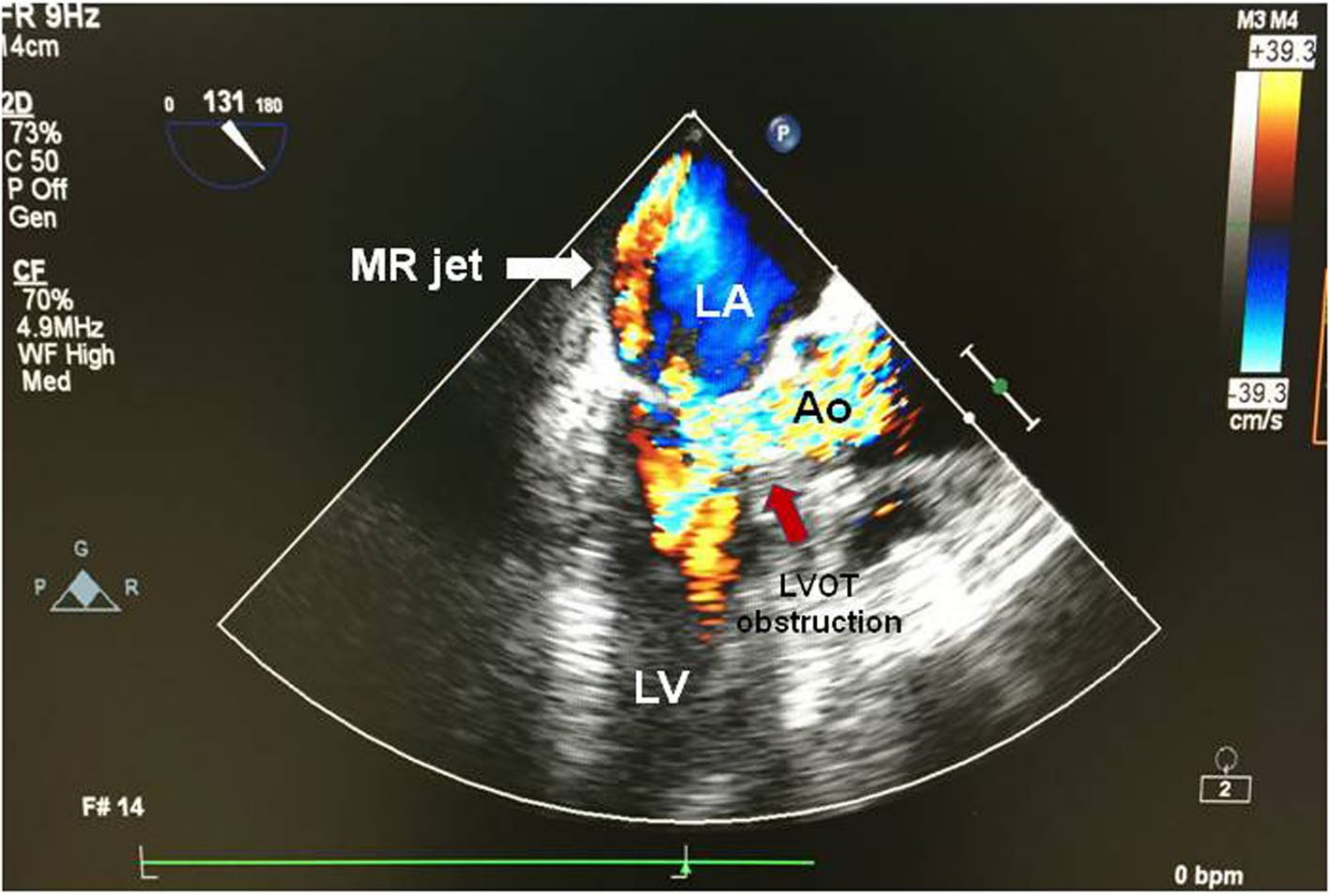
Intraoperative transesophageal echocardiography with color-flow Doppler mapping (midesophageal long axis view) showed a significant MR jet (white arrow) and mosaic flow signals in the left ventricular outflow tract (red arrow). LA = left atrium; LV = left ventricle; LVOT = left ventricular outflow tract; MR = mitral regurgitation; Ao = aorta. An additional movie file shows this in more detail (see Additional file 1)

At preoperative evaluation, he had good exercise tolerance without chest pain, syncope, shortness of breath or palpitations on exertion. Chest X-ray (CXR) revealed a normal cardiac silhouette and no pulmonary lesions. Electrocardiogram (ECG) showed normal sinus rhythm without left ventricular hypertrophy or left atrial enlargement. Laboratory studies, including blood chemistry and coagulation profile, were within acceptable limits. In the operating room, initial routine monitoring showed sinus rhythm with a heart rate (HR) of 76 beats per minute, blood pressure (BP) of 140/63 mmHg, respiratory rate of 15 per minute, and 94% pulse oxygen saturation on room air. After adequate hydration with intravenous administration of 5% dextrose and 0.45% sodium chloride, spinal anesthesia was achieved with an injection of 0.5% hyperbaric bupivacaine 9.5 mg and fentanyl 15 μg into the subarachnoid space via a 26-gauge spinal needle at the L3/L4 level. Surgery commenced when the sensory block reached the T10 dermatome (assessed by pinprick sensation). With spinal anesthesia, his BP was still relatively stable around at 120/60 mmHg, while his HR gradually increased to 90 beats per minute. One hour after anesthesia, the patient became uncomfortable and restless. His cardiac rhythm changed to atrial fibrillation with a moderate ventricular response of 100 beats per minute. He therefore received intravenous midazolam 0.5 mg and fentanyl 25 μg in an attempt to alleviate his distress, and intravenous digoxin 0.125 mg was administered for rhythm control. However, his condition did not improve, and he became acutely dyspneic with pink frothy secretions. 100% oxygen was administered via the face mask, but his oxygen saturation decreased to 88%, while his BP increased to 145/65 mmHg. Crackles were heard on chest auscultation. The impression at this stage was acute pulmonary edema or TURP syndrome. Therefore, intravenous furosemide 5 mg and sublingual nitroglycerin 0.6 mg were given. However, his systolic BP dropped to 87/50 mmHg, and he became less alert. Surgery was discontinued and general anesthesia was conducted with tracheal intubation. An arterial line was inserted and with a fraction of inspired oxygen at 50%, his arterial blood gas showed pH 7.21, pCO_2_ 46 mmHg, pO_2_ 83 mmHg, with severe metabolic acidosis, but no hyponatremia. He remained hypotensive and significant ST depression was noted. Thus, a titrated infusion of fluid and dopamine 5 μg/kg/min was initiated on suspicion of acute myocardial infarction. Unfortunately, his hypotension worsened and atrial fibrillation ventricular response rate increased to 160 beats per minute. Bedside TEE was promptly performed, which revealed a small hypercontractile left ventricle (LV), mosaic flow signal in the LVOT and a significant jet of MR (Fig. 1) (see Additional movie file 1), which are highly suggestive of SAM of the mitral valve, but his extreme tachycardia made it difficult to confirm this. Nevertheless, aggressive fluid resuscitation was administered, with improvement in his BP to 150/60 mmHg, and HR to 92 beats per minute. He was then transferred to the intensive care unit (ICU) for further evaluation.

**Fig. 2:**
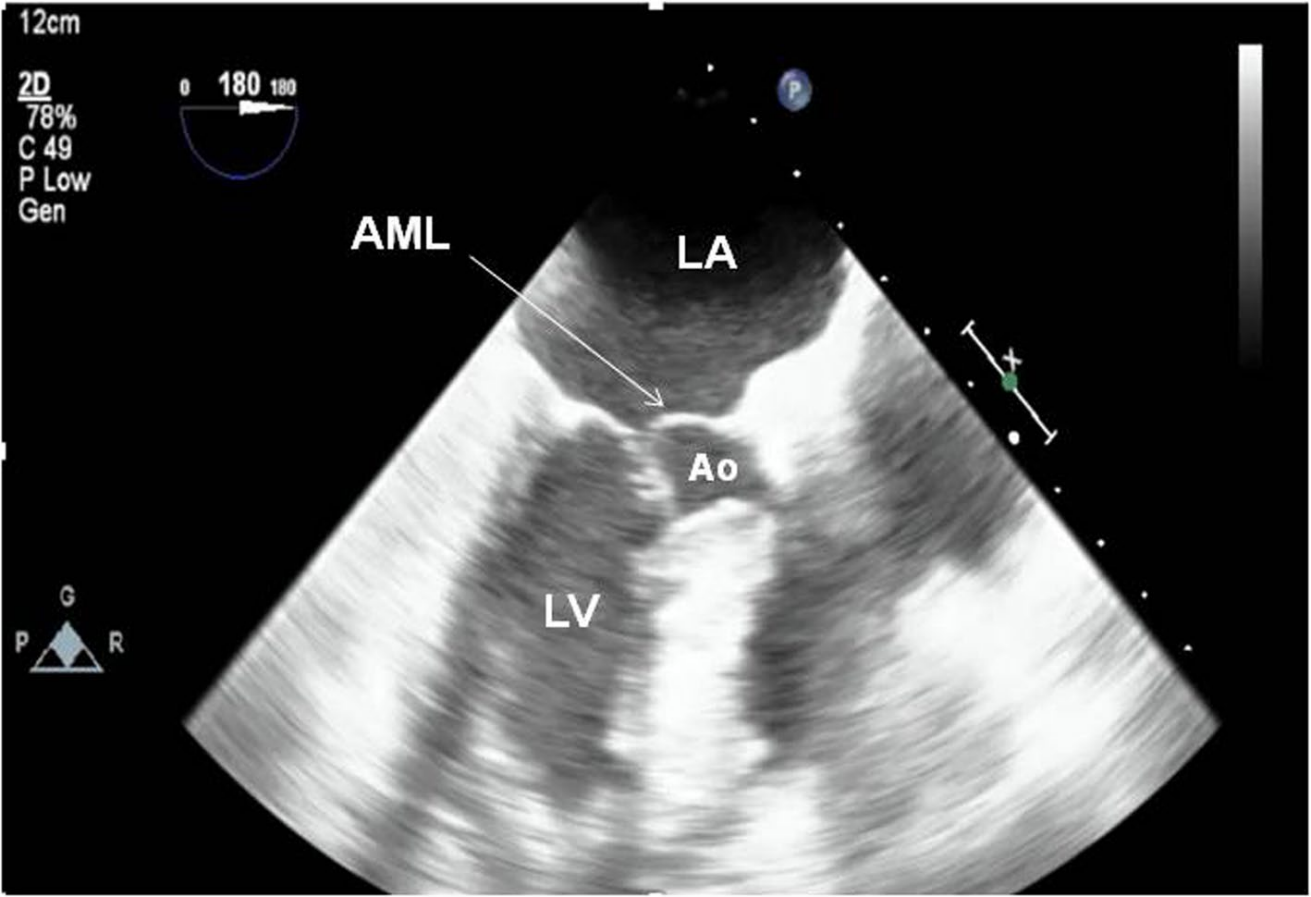
Postoperative transesophageal echocardiography in the intensive care unit (midesophageal long axis view) revealed systolic anterior motion of the anterior mitral leaflet into the left ventricular outflow tract. LA = left atrium; LV = left ventricle; Ao = aorta; AML = anterior mitral leaflet. An additional movie file shows this in more detail (see Additional file 2)

Upon arrival at ICU, CXR showed pulmonary infiltrates, and his serum troponin I level was elevated at 2.338 ng/mL (reference range < 0.1 ng/mL), suggesting mild myocardial injury. After he became hemodynamically stable, a repeat TEE showed mild concentric left ventricular hypertrophy, SAM of the anterior mitral leaflet into the LVOT with subaortic obstruction and resultant severe eccentric dynamic MR on color-flow Doppler mapping (Fig. 2) (see Additional movie file 2). LV systolic function was preserved without regional dyskinesia. He was weaned from the ventilator 7 days later and transferred to the general ward on day 9.

## Discussion and conclusions

SAM of the mitral valve is a relatively rare but potentially life-threatening phenomenon. It consists of the anterior movement of the mitral valve leaflets towards the LVOT during systole, causing LVOT obstruction and resultant MR, leading to reduction in cardiac output and hemodynamic collapse [1]. Typically, it is common in patients with HCM or may be a complication of certain cardiac surgeries, such as mitral valve repair and aortic valve replacement [2, 4, 5]. Conditions that cause cardiac septal wall motion abnormalities or changes in LV geometry, including diabetes mellitus, hypertensive heart disease, and acute myocardial infarction can sometimes result in this phenomenon as well [6]. In recent years, there have also been reports of SAM in the critical care setting in patients without apparent underlying structural cardiac abnormalities [3].

The pathophysiology of SAM remains unclear. The original hypothesis focuses on the Venturi effect through a narrowed LVOT caused by bulging of a hyperdynamic and hypertrophic septum in the hypovolemic state [3, 7]; however, recent investigations indicate that the predominant mechanism is through rapid blood flow velocities in the LVOT caused by LV hypercontractility, dragging the mitral valve leaflets anteriorly into the septum [8, 9]. While the main feature predisposing to SAM is a hypercontractile LV, primary anomalies of the mitral valve apparatus, HCM or an underfilled ventricle from reduction in LV preload can also provoke SAM. In the perioperative setting, various hemodynamic conditions such as anesthesia-mediated vasodilation either from general anesthetics or neuraxial anesthesia, increased catecholamine levels due to surgical stimulation or the use of inotropic medications, and absolute or relative hypovolemia from preoperative fasting or perioperative bleeding can all increase the risk and aggravate the severity of SAM, even in the absence of underlying cardiac disease [3, 10].

In our case, acute respiratory distress and restlessness during surgery were initially incorrectly treated as pulmonary edema from fluid overload, based on his clinical features (dyspnea, desaturation, and crackles on auscultation) and the assumption of excessive irrigation fluid absorption following TURP. In fact, TEE showed that this patient was hypovolemic, but he developed pulmonary edema from the combination of atrial fibrillation with tachycardia, reduced hypertrophic LV compliance, and MR secondary to SAM. We speculate that spinal anesthesia, which can substantially decrease peripheral vascular resistance and ventricular preload, may have predisposed him to develop SAM, when he became volume depleted. However, our patient’s presentation was not typical of SAM, as he was initially mildly hypertensive, but this may have been an adrenergic response to his dyspnea. The paradoxical clinical features contributed to the initial misconception that his hypoxemia was a result of pulmonary edema from fluid overload during TURP. The administration of furosemide and nitroglycerin, used for treatment of cardiogenic pulmonary edema, likely aggravated the severity of his hypoxemia caused by SAM-induced MR and worsened his hemodynamics.

Our case demonstrates that SAM may represent a potential cause of unexpected hypoxia and/or refractory hypotension in patients undergoing spinal anesthesia in the context of hypovolemia and adrenergic stimulation. In the critical care setting, echocardiography may be an important first-line diagnostic approach of SAM as it provides direct visualization of the underlying mechanic pathology, and can help differentiate this from other causes of severe cardiopulmonary events [11, 12]. Diagnostic features of SAM include the distal anterior mitral valve leaflet being displaced into the LVOT during systole with a turbulent flow in the LVOT on color Doppler, followed by a posterior-directed MR jet [1, 13]. Under continuous wave Doppler, a high-velocity flow profile with a delayed peak (“dagger-shaped” signal), which is related to dynamic LVOT obstruction, is observed while tracing blood flow through the LVOT [13]. In our patient, initial TEE revealed only the mosaic pattern in the LVOT with a significantly eccentric MR jet, but it was difficult to clearly assess the mitral valve movement, because of profound tachycardia. Nevertheless, it is important to note that these echographic features may be suggestive of SAM, when there is unexplained hypoxemia or hypotension in the perioperative period, even in patients without obvious underlying heart disease.

Bedside TEE plays an essential role not only for timely diagnosis of SAM but also in facilitating early treatment of underlying causative factors and thus preventing the development of SAM. The principles of medical therapy for SAM are to increase LV preload and reduce the hypercontractile state [8, 9]. Options include volume resuscitation and if required, vasopressor infusion (e.g. phenylephrine) to restore LV volume and prevent cardiovascular collapse. Our case indicates that fluid balance should be carefully monitored and aggressive management instigated for hypovolemia, particularly in urological patients having spinal anesthesia, as they usually undergo fluid restriction and/or routine diuretic therapy in the perioperative period. For patients in a hypercontractile state, cessation of vasodilators and inotropes can be helpful. Moreover, β-adrenergic blockers have been considered as an alternative treatment option in selective cases to decrease the HR, with prolongation of the LV filling period, and also reduce the force of contraction [3, 14].

In conclusion, SAM may result in unexpected perioperative cardiopulmonary deterioration, particularly in the presence of myocardial hypertrophy or in vulnerable situations such as hypovolemia and anesthesia-mediated vasodilatation. Our case highlights that SAM can be easily overlooked and may clinically resemble cardiogenic pulmonary edema when actually in the hypovolemic state, which might lead to incorrect treatment. Prompt echocardiography plays an essential role in making a timely diagnosis and guiding appropriate management.

## Supplementary Information

**Additional file 1.** (intraoperative TEE.mov): Intraoperative transesophageal echocardiography with color-flow Doppler mapping (midesophageal long axis view) showed a significant MR jet and mosaic flow signals in the left ventricular outflow tract.

**Additional file 2.** (postoperative TEE in the ICU.mp4): Postoperative transesophageal echocardiography in the intensive care unit (midesophageal long axis view) revealed systolic anterior motion of the anterior mitral leaflet into the left ventricular outflow tract.

## Data Availability

Not applicable.

## Abbreviations

SAM: Systolic anterior motion
LVOT: Left ventricular outflow tract
MR: Mitral regurgitation
HCM: Hypertrophic cardiomyopathy
TURP: Transurethral resection of the prostate
TEE: Transesophageal echocardiography
CXR: Chest X-ray
ECG: Electrocardiogram
HR: Heart rate
BP: Blood pressure
LV: Left ventricle
ICU: Intensive care unit

## References

[1] Sidebotham D, Legget M. The mitral valve. In: Sidebotham, editor. Practical and perioperative transesophageal echocardiography. Philadelphia: Butterworth-Heinemann; 2003. p. 149–50.

[2] Fujita Y, Kagiyama N, Sakuta Y, Tsuge M (2015). Sudden hypoxemia after uneventful laparoscopic cholecystectomy: another form of SAM presentation. BMC Anesthesiol.

[3] Luckner G, Margreiter J, Jochberger S, Mayr V, Luger T, Voelckel W (2005). Systolic anterior motion of the mitral valve with left ventricular outflow tract obstruction: three cases of acute perioperative hypotension in noncardiac surgery. Anesth Analg.

[4] Brown ML, Abel MD, Click RL, Morford RG, Dearani JA, Sundt TM (2007). Systolic anterior motion after mitral valve repair: is surgical intervention necessary?. J Thorac Cardiovasc Surg.

[5] Routledge T, Nashef SA (2005). Severe mitral systolic anterior motion complicating aortic valve replacement. Interact Cardiovasc Thorac Surg.

[6] Haley JH, Sinak LJ, Tajik AJ, Ommen SR, Oh JK (1999). Dynamic left ventricular outflow tract obstruction in acute coronary syndromes: an important cause of new systolic murmur and cardiogenic shock. Mayo Clin Proc.

[7] Maron BJ, Bonow RO, Cannon RO, Leon MB, Epstein SE (1987). Hypertrophic cardiomyopathy- interrelations of clinical manifestations, pathophysiology, and therapy. N Engl J Med.

[8] Ibrahim M, Rao C, Ashrafian H, Chaudhry U, Darzi A, Athanasiou T (2012). Modern management of systolic anterior motion of the mitral valve. Eur J Cardiothorac Surg.

[9] Raut M, Maheshwari A, Swain B (2018). Awareness of ‘systolic anterior motion’ in different conditions. Clin Med Insights Cardiol.

[10] Cavallaro F, Marano C, Sandroni C, Dell'anna A (2010). Systolic anterior motion causing hemodynamic instability and pulmonary edema during bleeding. Minerva Anestesiol.

[11] Reddy S, Ueda K (2014). Unexpected refractory intra-operative hypotension during non-cardiac surgery: diagnosis and management guided by trans-esophageal echocardiography. Indian J Anaesth.

[12] Hori K, Matsuura T, Mori T, Nishikawa K (2015). Usefulness and growing need for intraoperative transthoracic echocardiography: a case series. BMC Anesthesiol.

[13] Hymel BJ, Townsley MM (2014). Echocardiographic assessment of systolic anterior motion of the mitral valve. Anesth Analg.

[14] Hertel T, Banayan JM, Chaney MA, von Dossow V, Dhawan R (2017). Systolic anterior motion of the mitral valve with left ventricular outflow tract obstruction: a rare cause of hypotension after lung transplantation. J Cardiothorac Vasc Anesth.

